# Instantaneous physico-chemical analysis of suspension-based nanomaterials

**DOI:** 10.1038/srep09896

**Published:** 2015-04-29

**Authors:** Fanxu Meng, Victor M. Ugaz

**Affiliations:** 1 Artie McFerrin Department of Chemical Engineering; 2 Department of Biomedical Engineering Texas A&M University College Station, Texas, U.S.A

## Abstract

High-throughput manufacturing of nanomaterial-based products demands robust online characterization and quality control tools capable of continuously probing the in-suspension state. But existing analytical techniques are challenging to deploy in production settings because they are primarily geared toward small-batch *ex-situ* operation in research laboratory environments. Here we introduce an approach that overcomes these limitations by exploiting surface complexation interactions that emerge when a micron-scale chemical discontinuity is established between suspended nanoparticles and a molecular tracer. The resulting fluorescence signature is easily detectable and embeds surprisingly rich information about composition, quantity, size, and morphology of nanoparticles in suspension independent of their agglomeration state. We show how this method can be straightforwardly applied to enable continuous sizing of commercial ZnO nanoparticles, and to instantaneously quantify the anatase and rutile composition of multicomponent TiO_2_ nanoparticle mixtures pertinent to photocatalysis and solar energy conversion.

Nanomaterials have long functioned as key additives in a broad array of familiar products (e.g., paints, sunscreens, composites), but their significance has grown considerably in recent years as their exceptional electrical, optical, and mechanical properties become harnessed in sophisticated new ways (e.g., photovoltaics, catalysis, sensors^1^,^2^,^3^,^4^,^5^,^6^,^7^). Many of these applications involve processing of suspension-based nanomaterials and demand precise control over properties such as chemistry, size, morphology and/or crystalline structure (e.g. anatase vs. rutile)^2^,^3^,^8^,^9^,^10^. Unfortunately, characterization methods have generally failed to keep up with the rapid pace of material discovery. Measurement approaches such as dynamic light scattering (DLS) and electron microscopy (SEM, TEM)—workhorses in the field for decades—are challenging to employ outside of research-oriented laboratory settings and do not easily lend themselves to continuous analysis because of their sample requirements (dry powder, dilution), operating conditions (high vacuum), and measurement duration (tens of minutes). Sizing results are also often sensitive to the presence of agglomerates and aggregates of primary particles. This lack of continuous characterization tools scalable toward online deployment, particularly methods capable of directly probing the in-suspension state to simultaneously obtain size and species information (e.g., to support continuous nanomaterial synthesis^11^,^12^,^13^,^14^,^15^,^16^,^17^,^18^), has made it challenging to establish standardized manufacturing-scale quality control benchmarks and therefore imposes a significant bottleneck between scientific discovery and commercialization^19^.

Here we introduce an approach that overcomes limitations of conventional small-batch analytical methods, enabling continuous online quantification and characterization of nanoparticle composition, size, and morphology, directly in suspension and independent of agglomeration state. Our method exploits surface complexation interactions that emerge when a sharp (micron-scale) chemical discontinuity is established between suspended nanoparticles and a molecular tracer in a laminar flow environment that removes limitations associated with convective transport and mixing (Fig. 1a). The resulting interfacial fluorescence signature is easy to detect and embeds surprisingly rich information about particle species (via the nature of fluorescence enhancement or quenching), size (via the relative magnitude of the fluorescence signature), and their combined concentration dependence. The extent of fluorescence enhancement/quenching and lateral shift of the interface between co-flowing nanoparticle and tracer streams are observables that, when supplied as inputs to a physico-chemical model we describe here, make it possible to instantly obtain physical parameters associated with the suspended nanomaterials from a single convenient *in-situ* snapshot measurement (Fig. 1b).

## Results

### Species, concentration, and size dependence

**Figure 1:**
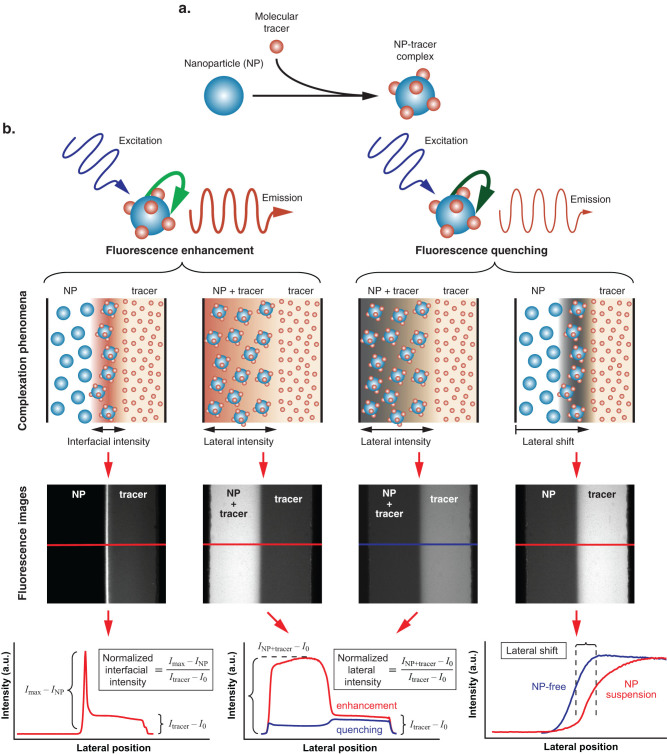
Analysis and quantification of fluorescent complexation. (a) Distinct fluorescence signatures emerge from surface complexation between nanoparticles and a molecular tracer. (b) These phenomena can be continuously observed by establishing a sharp (micron scale) gradient between adjacent nanoparticle and tracer streams in a microscale laminar flow environment (flow direction is vertical). Nanoparticle size and concentration information is embedded in the interfacial and lateral features of the corresponding fluorescence images. The intensity profiles can be quantified in multiple ways depending on the strength of the fluorescence signal and whether enhancement or quenching are observed. Illustrative diagrams are qualitative and not drawn to scale.

**Figure 2:**
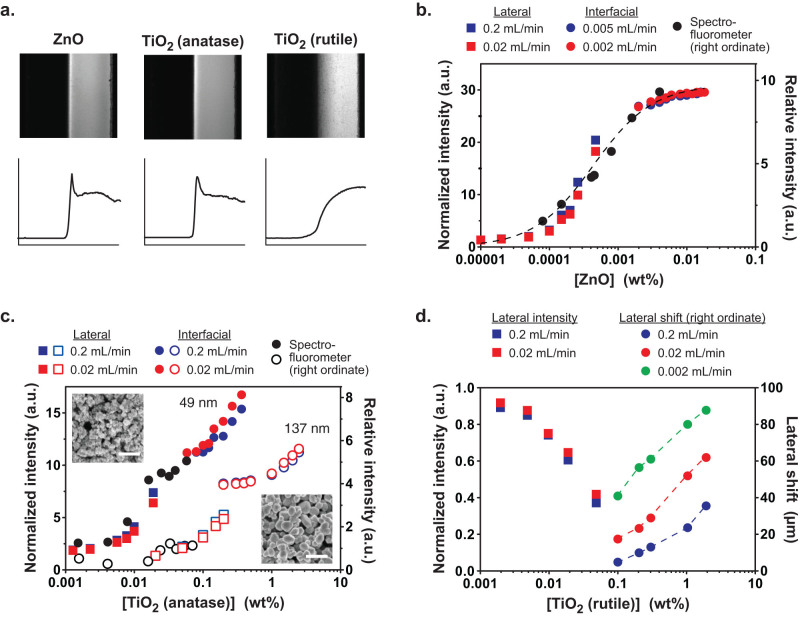
Fluorescent complexation sensitively depends on nanoparticle concentration, species, size, and morphology. (a) The material-dependence of interfacial fluorescence becomes evident upon comparison of ZnO (60 ± 20 nm) and TiO_2_ (anatase: 49 ± 9 nm; rutile: 137 ± 36 nm). Left half of image: 50 mM nanoparticle suspension; right half of image: 0.033 mg mL^−1^ fluorescein tracer. Upper panels show images of co-flowing streams, lower panels show the corresponding lateral intensity profile (microchannels are 500 μm wide). (b) Broad quantitative sensitivity over a span of 4 orders of magnitude of nanoparticle concentration is attained by combining data from interfacial and pre-mix approaches (dashed line connecting the spectrofluorometer data points is included to guide the eye, a constant vertical shift factor was applied to align the lateral and interfacial data to clearly depict the concentration dependent trend). (c) Fluorescent complexation is sensitively dependent on nanoparticle size, enabling differences to be distinguished independent of agglomeration state (insets show SEM images of nanoparticle powders, bar 400 nm). (d) Morphological sensitivity is evident by transformation from fluorescence enhancement in anatase TiO_2_ to quenching in rutile TiO_2_, where characterization in terms of the interfacial shift distance enables the accessible concentration range to be greatly extended (dashed lines connecting the shift distance data are included to guide the eye). Particle sizes were obtained by analysis of SEM data, [Tracer] = 0.0165 mg mL^−1^ in (b), (c), and (d).

To illustrate how this interfacial signature depends on properties of suspended nanomaterials, we characterized interactions between ZnO (60 ± 20 nm) and TiO_2_ (anatase: 49 ± 9 nm and 137 ± 36 nm, rutile: 40 ± 7 nm) nanoparticles with a fluorescein tracer. Fluorescence enhancement is observed in ZnO and anatase TiO_2_, whereas quenching is observed in rutile TiO_2_ (Fig. 2a). In the case of ZnO, a strong concentration dependence can be resolved over 4 orders of magnitude in nanoparticle concentration (Fig. 2b). This wide sensitivity range is made possible by combining interfacial (optimal at higher concentrations where a distinct local interfacial signature is clearly evident) and lateral (optimal at low concentrations where pre-mixing the particles and tracer generates a stronger signal that can be measured across a larger region of interest) fluorescence intensity data (Fig. 1b), yielding results consistent with conventional bulk spectrofluorometer measurements. The underlying complexation phenomena reflect interactions between Zn^2+^ ions in the ZnO matrix and carbonyl groups in the tracer^20^. The fluorescence signatures are also dependent on particle size, as seen by comparison of data from suspensions containing 49 and 137 nm anatase TiO_2_, where a 4-fold intensity increase is observed in the smaller diameter material (Fig. 2c). Enhanced sensitivity to smaller particle sizes is a unique feature of our approach, and reflects the inherently surface-dominated complexation mechanism that is most pronounced at the smallest particle sizes where the surface area to volume ratio is maximized^21^. Remarkably, this size dependent sensitivity is achievable regardless of the material's agglomeration state, as can be seen upon comparison with DLS data suggesting that the characteristic diameters of both materials are > 200 nm (Methods). This discrepancy emerges because scattering techniques are unable to clearly discern clustering of small-sized nanoparticles into agglomerates with dimensions comparable to individual larger nanoparticles (SEM insets in Fig. 2c). Our approach also reveals isoforms in crystalline morphology, as demonstrated in the case of TiO_2_ where fluorescence enhancement observed in anatase samples transforms to quenching in the rutile isoform (Fig. 2d).

### Physico-chemical characterization

**Figure 3:**
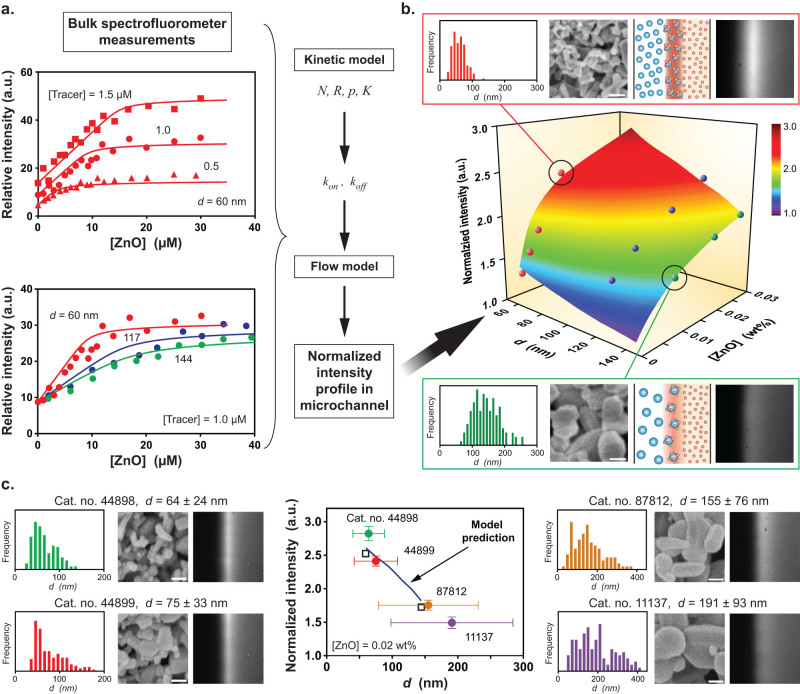
ZnO nanoparticle size and concentration information are embedded in the interfacial fluorescence signature. (a) Bulk spectrofluorometer measurements at different tracer concentrations are first performed using 60 ± 20 nm particles followed by subsequent measurements as a function of particle size using 117 ± 36 and 144 ± 41 nm nanoparticles to obtain kinetic parameters. (b) These parameters serve as inputs to a flow model that captures the combined effect of particle size and concentration on the fluorescence signature (normalized interfacial intensity) measured in a microchannel experiment ([Tracer] = 5 µM). This format permits online characterization of nanomaterials under continuous flow. Insets show SEM images and corresponding size distributions of nanoparticle powders (bars, 100 nm) along with the observed fluorescence profile (microchannels are 500 μm wide). (c) The surface plot in (b) enables instantaneous sizing of 4 different aqueous ZnO suspensions prepared from commercial nanopowders based on interfacial intensity measurements (Alfa Aesar; catalog numbers and size distributions from SEM analysis are shown in each panel beside the interfacial fluorescence profiles; size scales are the same as in (b)). Vertical error bars represent the standard deviation of 3 independent microchannel-based fluorescence measurements, horizontal error bars represent the standard deviation of corresponding SEM particle size measurements (*n* > 100). Open squares show model predictions that represent polydispersity effects by considering the full ensemble of coupled multiple dye-nanoparticle interactions across the entire size distribution for the 60 and 144 nm materials in (a).

We developed a physical model that captures the interplay among particle species, size, and concentration governing these fluorescence signatures (Fig. 3). Our model is based on considering the number of available surface binding sites on the suspended nanoparticles [N_S_], which is in turn related to the nanomaterial's molar concentration [M]_i_ via [N_s_] = *N* [M]_i_ (*N* expresses the moles of binding sites per mole of nanomaterial; i.e., binding sites per mole of nanoparticle/moles of nanomaterial per mole of nanoparticle). At equilibrium, these complexation interactions can be represented by a stoichiometric balance of the form 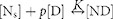 where *p* represents the number of fluorescein molecules associated with each surface binding site and [ND] is the molar concentration of sites occupied by dye-nanoparticle complexes. The equilibrium constant *K* is expressed in terms of association and disassociation steps as 
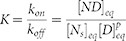
, where the surface sites available for complexation are represented by [N_s_]_eq_ = *N* [M]_i_ − [ND]_eq_ and the free dye available for complexation is determined from [D]_eq_ = [D]_i_ − *p*[ND]_eq_. Combining these expressions enables the nanoparticle concentration in suspension [M]_i_ to be correlated with the observed fluorescence signature via its dependence on [ND]_eq_ (Methods).
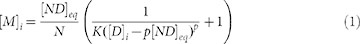
The constants *N*, *K*, *p*, and *R* (a parameter expressing the relative fluorescence intensity of the free and bound tracer, Methods) are determined by fits to data obtained from a series of bulk spectrofluorometric measurements as a function of tracer and nanoparticle concentration (Fig. 3a). The equilibrium constant *K* is then used to guide selection of *k_on_* and *k_off_* values employed in a reaction-diffusion flow model that quantitatively predicts the fluorescence signatures observed in our microchannel-based experiments. In the case of binary mixtures, where the same species is present in two different particle sizes, the fluorescence response can in principle be uniquely determined if the total mass of solids in suspension is held constant by leveraging the dependence of fluorescence on surface area, where the total fluorescence is represented as a linear combination of contributions from each species. Although uniqueness becomes more challenging to guarantee beyond two components, this is not expected to impose a major limitation in the context of envisioned applications of our approach to enable online characterization in settings involving manufacture of nanomaterials whose size and properties fall within a relatively well established envelope.

We applied this framework toward analysis of ZnO nanoparticles, enabling the interdependence among particle size, species, and concentration to be expressed in terms of a surface plot construction uniquely relating experimentally measured fluorescence to the compositional state of the suspended nanomaterials (Fig. 3b). This fingerprint makes it possible to instantaneously extract size and/or concentration information from unknown samples via a single microchannel-based fluorescence measurement. To illustrate how this capability could be implemented in a setting relevant to manufacturing, we measured interfacial fluorescence intensities of aqueous suspensions prepared from four different commercial ZnO nanopowders (Fig. 3c). Particle sizes ranged from 64 to 191 nm, and suspension concentrations were held constant at 0.02 wt%. Fluorescence data obtained in the microchannel format yield mean particle sizes that agree remarkably well with the model prediction in Fig. 3b, especially considering the inherently heterogeneous particle size distributions and the assumptions involved in our model formulation. Our approach therefore makes it straightforward to continuously monitor nanoparticle size directly in suspension, enabling routine online characterization.

We remark that polydispersity effects are inherently embedded into our model by virtue of the fact that its parameters (*N*, *K*, *p)* are obtained from bulk spectrofluorometer measurements in (Fig. 3a). Although strictly speaking these parameters may be size dependent, this is a reasonable compromise in the absence of monodisperse calibration samples, and in consideration of our envisioned characterization applications where sharply bi-modal or multi-modal size distributions are not anticipated (i.e., our method is highly amenable to enable rapid online analysis in manufacturing settings where properties are not expected to significantly deviate beyond a well-defined window). To validate this hypothesis, we simulated the fluorescence response from an ensemble of particles binned to mimic the size distributions of 60 and 144 nm nanoparticle samples employed to obtain kinetic model parameters (size distributions and SEM data shown in Fig. 3b). The full ensemble of coupled multiple dye-nanoparticle reactions involving each particle size in the distribution was evaluated and combined to generate the collective fluorescence signature (see Supplementary Table S1b for parameters applied in the computational model). The results of this analysis (open square symbols in Fig. 3c) display close agreement with our initial model predictions where polydispersity was not explicitly included (solid line in Fig. 3c). Since our model is effectively “trained” using samples displaying size distributions similar to those expected to be encountered in its practical application, it is therefore reasonable to expect that large deviations from the resulting predictions are unlikely under conditions the particle size distribution profiles display similar characteristics.

### Analysis of multi-component systems

**Figure 4:**
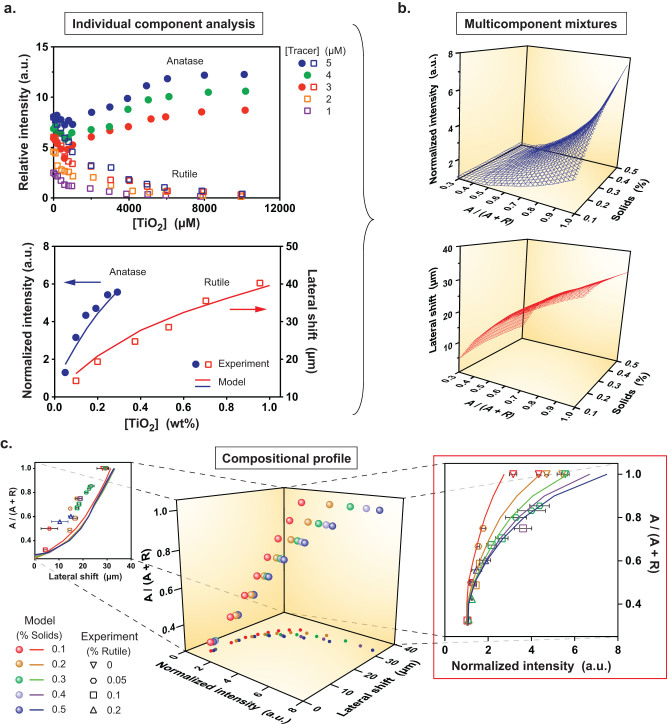
Quantitative composition analysis of anatase and rutile TiO_2_ nanoparticle mixtures. (a) Kinetic parameters are obtained from the bulk fluorescence response displayed by each nanoparticle species (anatase: enhancement, 49 ± 9 nm, rutile: quenching, 40 ± 7 nm), enabling the corresponding fluorescence (normalized interfacial intensity, lateral shift) measured in microchannel experiments to be predicted. (b) The flow model is then used to map these fluorescence signatures for anatase and rutile mixtures over an ensemble of compositions (expressed in terms of the anatase mass fraction, anatase/(anatase + rutile) = A/(A + R), [Tracer] = 5 µM). (c) These data are then cross-plotted so that the anatase fraction can be uniquely determined from simultaneous normalized interfacial intensity and lateral shift measurements over a range of compositional states (e.g., % solids and % rutile in suspension, symbols are color-coded to indicate corresponding % solids, experiment data are binned as mean ± 0.05% (i.e., 0.1% solids includes data binned from experiments ranging from 0.05 to 0.149% solids, etc.). Interfacial intensity predictions agree with experiment data (right), whereas the lateral shift is not strongly composition dependent over the range of conditions explored here (left).

A manufacturing scenario of particular importance involves ensuring that consistent relative amounts of anatase and rutile species are maintained in multicomponent mixtures of TiO_2_ nanoparticles. Our method can be readily adapted to perform this characterization in a continuous format by exploiting the distinct fluorescence signatures displayed by each component (Fig. 2). Although nanoparticle-tracer complexation occurs via a similar pathway in each species, differences in the band gap between energy levels lead to fluorescence enhancement in the anatase form and quenching in the rutile species^22^,^23^. The relative quantity of each isoform present in the mixture can thus be determined by simultaneous analysis of interfacial intensity and lateral shift information (subject to the constraint that each species displays a sufficiently different fluorescence enhancement or quenching signature, as is the case with TiO_2_), where the same framework we developed for ZnO nanoparticles is applied to evaluate kinetic parameters (Fig. 4a and Methods). Our model successfully captures the observed trends of increased intensity and reduced lateral shift with increasing fraction of anatase TiO_2_ in the overall solid content, enabling the fluorescence intensity and lateral shift to be predicted across a range of compositional states (Fig. 4b, compositions are expressed in terms of the anatase mass fraction, anatase/(anatase + rutile) = A/(A + R)). Cross-plotting these data then enables the anatase fraction to be uniquely determined from these simultaneously measured observables (Fig. 4c). Precise control over the compositional profile of mixed-phase TiO_2_ nanoparticles is a critical factor governing photocatalytic reaction performance^9^, solar conversion efficiency^24^, and toxicity^25^. Notably, many of these applications involve mixtures containing optimal anatase fractions ranging from 0.75 ~ 0.9^9^,^24^,^26^, well within the analytical range of our method. The lateral shift does not display a strong composition dependence under these conditions, leaving interfacial intensity as the primary quantity of interest. Our model displays good agreement with experimentally measured values of interfacial intensity spanning a range of compositional states (Fig. 4c), enabling detailed *in-situ* characterization information to be continuously extracted from microchannel-based fluorescence measurements.

## Discussion

Compared with conventional characterization based on x-ray diffraction^9^,^27^, a batch technique requiring dry powder samples, the speed and simplicity of our continuous *in-situ* approach combined with its ability to provide both size and compositional information offers compelling advantages for routine compositional monitoring in nanomanufacturing settings (e.g., to ensure that material properties are maintained within clearly defined limits). Test samples can be individually analyzed from larger product batches, and continuous monitoring of interfacial fluorescence can be performed without compromising material purity by positioning an outlet to collect the un-complexed nanoparticle effluent. Our methodology is inherently versatile and can be readily applied to establish fluorescence signature fingerprints of complex (and more realistic) suspension-based nanomaterial products incorporating coatings and stabilizing additives. Species-specific labeling and establishment of fluorescence “fingerprint” libraries can enable analysis of more complex materials and mixtures. Few currently available techniques are able to instantaneously deliver this kind of quantitative characterization in an online format, suggesting broad applicability as a routine tool that can supplement the workhorse analytical methods in a host of emerging manufacturing settings.

## Methods

### Physico-chemical model

A primary consideration in the development of our model involves quantifying how the available surface adsorption sites (a key parameter governing fluorescent complexation) are related to the bulk oxide concentration in suspension (the most convenient observable quantity). We accomplish this by expressing surface binding site concentration [N_S_] is terms of the nanomaterial's molar concentration [M]_i_ via [N_s_] = *N* [M]_i_ (*N* expresses the binding sites per mole of nanomaterial; i.e., moles of surface binding sites per nanoparticle/moles of nanomaterial per nanoparticle). Since most of the nanomaterial is “buried” within the particle interior, it follows that *N* should be ≪ 1. It can be inferred that the parameter *N* increases as nanoparticle size decreases due to the higher surface area to volume ratio, thereby providing an indirect indication of the specific surface area.

At equilibrium, fluorescent complexation can be represented by a stoichiometric balance of the form 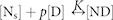, where *p* represents the number of dye molecules associated with each surface binding site and [ND] is the molar concentration of sites occupied by dye-nanoparticle complexes. The equilibrium constant *K* is expressed in terms of association and disassociation steps as 
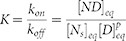
, where the surface sites available for complexation are represented by [N_s_]_eq_ = *N* [M]_i_ − [ND]_eq_ and the free dye available for complexation is obtained from [D]_eq_ = [D]_i_ − *p*[ND]_eq_. Making these substitutions yields.
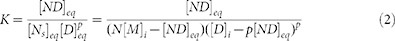
Equation (2) can be rearranged to obtain an expression for [ND]_eq_ as a function of [M]_i_ and [D]_i_, as well as constants *N* and *K*. In this way, the nanoparticle concentration in suspension [M]_i_ can be correlated with the observed fluorescence signature via its dependence on [ND]_eq_ if the initial dye concentration [D]_i_ is known, as shown in equation (1).

The parameters *N*, *K*, and *p* are obtained by performing bulk spectrofluorometer measurements under equilibrium conditions to quantify the dependence of the observed fluorescence intensity *F_obs_* across an ensemble of dye and nanoparticle concentrations. To accomplish this, the fluorescence is decomposed into contributions from the free dye (*F_D_*) and bound nanoparticle-dye complexes (*F_ND_*) via *F_obs_* = *F′_D_* + *F′_ND_*. The absolute fluorescence variables are more conveniently expressed in terms of scaled quantities *F′_D_* = *F_D_* ([D]_eq_/[D]_i_) and *F′_ND_* = *F_ND_* (*p* [ND]_eq_/[D]_i_) reflecting the observation that [ND]_eq_ = 0 and *F_obs_* = *F_D_* when the sample contains only tracer, whereas 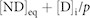 and *F_obs_* = *F_ND_* when all the dye is complexed with nanoparticles. Applying the stoichiometric relationship between [D]_eq_ and [ND]_eq_ yields.
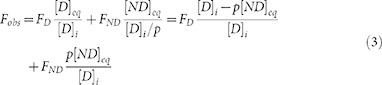
Upon rearranging and solving for [ND]_eq_, we obtain.
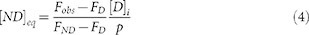
It is convenient to combine the free and complexed dye contributions by expressing them in terms of the ratio *R* = *F_ND_*/*F_D_*. Finally, substituting equation (4) into (1) and incorporating the definition of *R* enables the observed fluorescence *F_obs_* to be related to the concentration of suspended nanoparticles [M]_i_.
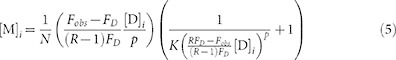
The constants *N*, *K*, *R*, and *p* in equation (5) are obtained from fits to data from a series of bulk spectrofluorometric measurements as a function of tracer dye and nanoparticle concentration. The results of applying this analysis to 60 ± 20 nm ZnO nanoparticles are shown in Fig. 3a. The Matlab function *nlinfit* was applied to simultaneously determine the parameter set of *N*, *K*, and *R* using an iterative least squares process, yielding the best simultaneous fit to data obtained at three different tracer dye concentrations for values of *p* ranging from 1 to 8 (Supplementary Fig. S1). For example, at *p* = 5 with initial guesses of *K* = 4,000, *N* = 0.015, and *R* = 4, output values of *K* = 4,240, *N* = 0.0161, *R* = 3.88 were obtained. Selection of an appropriate *p* value was guided by a desire to attain a realistic value of the fitted coefficient *N* in the vicinity of 0.02 or less (e.g., when *N* = 0.02, the surface density of effective binding sites = 8.3 sites/nm^3^, exceeding realistic density limits for metal oxide nanoparticles^28^). Consequently, we applied *p* = 5 for all subsequent analysis of ZnO, corresponding to a value of *N* = 0.016. This choice is supported by previous studies involving polymer interactions with alumina surfaces that have demonstrated how adsorption of multiple polymer functional groups per active site on the alumina surface can be favored under conditions leading to reduced intermolecular repulsion^29^. Although similar phenomena have been considered in previous studies, two important assumptions are generally applied in order to simplify the analysis^30^. First, comparable fits are often performed using the bulk oxide concentration, leading to values of *N* that are not consistent with the physically possible density of effective adsorption sites. Second, saturation conditions are assumed whereby the quantity of available binding sites is much greater than the number of available dye molecules. Our analysis is not subject to these simplifications, leading to binding constants that differ somewhat from other reported values.

We next extended the results obtained for 60 nm particles to enable analysis of spectrofluorometric measurements obtained from 117 ± 36 and 144 ± 41 nm ZnO nanoparticles (Fig. 3b). The value of *R* is assumed to remain constant because the nature of the chemical interactions associated with particle-tracer complexation is fundamentally unchanged for the same combination of nanomaterial and dye. The coefficient *N* is determined by scaling the value obtained at 60 nm to other particle sizes via its dependence on the surface to volume ratio. Equation (5) can then be applied to fit the concentration dependent fluorescence data at *p* = 5 to determine the equilibrium constant *K*, which in turn guides selection of the kinetic parameters *k_on_* and *k_off_* corresponding to each particle size^31^. Parameters obtained in our analysis of ZnO nanoparticles (Fig. 3) are given in Supplementary Table S1a. Effects of polydispersity in particle size could be incorporated using monodisperse test samples.

The same general procedure was followed for analysis of anatase and rutile TiO_2_ mixtures, however parameter selection was constrained by our desire to maintain the same particle size between species. Additionally, the concentration dependent trends observed in the spectrofluorometer data (initial decrease at low concentration in anatase, and quenching in rutile) incorporate additional complexities not fully captured by the framework in equation (5). We therefore selected a representative parameter set based on insights from our previous analysis for the purpose of evaluating the ability to characterize multicomponent mixtures. Parameters obtained in our analysis of TiO_2_ nanoparticles (Fig. 4) are given in Supplementary Table S2.

### Flow model

The kinetic parameters *k_on_* and *k_off_* are used as inputs for a flow model to predict the interfacial fluorescence intensity profiles observed in our microchannel-based experiments (i.e., Fig. 1). Following the framework developed by Yager et. al. for analysis of microfluidic immunoassays^32^,^33^,^34^,^35^,^36^, we constructed a 2D flow model using COMSOL Multiphysics to solve the steady-state Navier-Stokes equations (representing the flow field) simultaneously with a system of partial differential equations expressing coupled convection-diffusion processes with surface reactions (representing nanoparticle-tracer complexation).
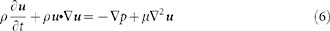

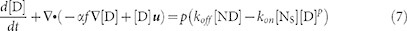

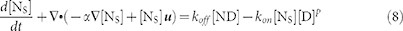

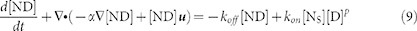


Fluid properties were assumed to be those of water at room temperature, and size-dependent nanoparticle diffusivity coefficients were estimated using the Stokes-Einstein relationship as *α* = *k_B_T*/(3π*µd*), where an average value of *d* representative of the size range of interest was chosen. Boundary conditions and variables are listed in Supplementary Tables S3 **and** S4.

### Nanomaterials and suspension preparation

Aqueous suspensions were prepared by dispersing commercial nanoparticle powders in deionized water, followed by 20 s of agitation using a digital vortex mixer (cat. no. 02215370; Fisher Scientific). All nanoparticle powders were used as received from the manufacturer in order to assess the capability of analyzing commercial samples. Materials employed in these studies are summarized in Supplementary Table S5.

### Microfluidic device construction and assembly

Y-shaped microchannels (40 µm tall, 500 µm wide, 2.4 cm long) were constructed in poly(dimethyl siloxane) (PDMS) using standard soft lithography. Master molds were prepared by spin coating SU-8 2025 photoresist onto silicon wafers, followed by a standard soft bake, UV exposure through the transparency film via a mask aligner, and development of the imprinted pattern. A freshly prepared PDMS mixture (10:1 volume ratio of base to crosslinker; Sylgard 184; Dow Corning) was degassed under vacuum and poured over the master mold to cast the microchannel structures. After curing at 80°C for 2 h, the mold was cooled to room temperature and individual microchannels were peeled away. Inlet and outlet holes were punched using a syringe needle, and the PDMS structures were bonded to glass microscope slides after O_2_ plasma treatment in a reactive ion etcher. Polyethylene tubing was inserted into the inlet and outlet holes to make fluidic connections.

### Spectrofluorometer experiments

Steady-state emission spectra of nanoparticle suspensions, dye solutions, and multicomponent mixtures were measured using a PTI QuantaMaster series spectrofluorometer. A xenon arc lamp (490 nm peak wavelength) was warmed up to 75 watts and steady state excitation was applied to the sample via a 495 nm long-pass filter. A digital emission scan from 500 to 800 nm was used with 1 nm step size and 0.1 second integration. 1–2 mL samples were loaded in cuvettes for analysis. Peak values of the intensity versus emission wavelength profiles were acquired and are expressed as *relative intensity* in bulk spectrofluorometer data.

### Microdevice operation and image acquisition

Continuous analysis was performed by co-injecting nanoparticle suspensions and the fluorescein tracer into the inlets of a y-shaped microchannel using a syringe pump (Model KDS-230, KD Scientific Inc.) at flow rates ranging from 0.002 to 0.2 mL/min (0.02 mL/min was used unless otherwise indicated). Images were analyzed to obtain descriptors of interfacial fluorescence phenomena summarized in Fig. 1 of the main text. Imaging was performed along the mid-plane of the microchannel to minimize sidewall effects. A flow rate of 0.02 mL/min (corresponding average velocity of 0.033 m/s) and 1.2 cm downstream image acquisition position yields characteristic residence times in the microchannel in the vicinity of 0.36 s.

### Scanning electron microscopy

Scanning electron microscopy (SEM) images of the nanoparticles were obtained using a JEOL JSM-6400 at an accelerating voltage of 5 keV and 7.5 mm working distance with SEI detector. Samples were prepared from dried suspensions. Images were taken with a 100 nm scale bar. To determine the nanoparticle size, each distinguishable nanoparticle was circumscribed and its size was calculated by circle area. Ensembles of at least 100 particles were analyzed to give size distributions. Size distribution data for the ZnO and TiO_2_ materials used in the main text are provided in Supplementary Figure S2.

### Dynamic light scattering

Dynamic light scattering (DLS) measurements were performed using a ZetaPALS instrument with a BI-9000AT correlator (Brookhaven Instruments Corp.). Anatase nanoparticles were dispersed in DI water at concentrations ranging from 0.002 to 0.03 wt%. Time-averaged particle size distributions were collected over an analysis period of at least 5 min at room temperature. Three separate measurements were acquired for each freshly prepared solution. The wavelength of the incident laser beam (λ) was 660 nm, and the detector angle (θ) was 90°. Autocorrelation functions were deconvoluted using the built-in nonnegatively constrained least squares-multiple pass (NNLS) algorithm in order to obtain particle size distribution. Particle sizes measured with DLS were considerably higher than those determined via SEM analysis, likely due to the presence of agglomerates in the samples. Supplementary Figure S3 shows representative data for anatase TiO_2_ nanoparticles with 49 ± 9 nm mean diameter (Cat. no. 637254, Sigma-Aldrich), where the DLS analysis reports a mean size of 330 nm. Similar results were obtained with all nanomaterials tested.

## Author Contributions

F.M. and V.M.U. designed the research project. F.M. performed the research and analyzed the data. F.M. and V.M.U wrote the manuscript.

## Supplementary Material

Supplementary Information Supplementary Information
