# Supplementary material for: Instantaneous physico-chemical analysis of suspension-based nanomaterials
Source: Sci Rep. 2015 Apr 29;5:9896. doi: 10.1038/srep09896 (PMC4413878; doi:10.1038/srep09896)
Supplement: Supplementary Information [file srep09896-s1.pdf]

Supplementary Information

Instantaneous physico-chemical analysis of suspension-based nanomaterials

Fanxu Meng and Victor M. Ugaz

Artie McFerrin Department of Chemical Engineering
Texas A&M University
College Station, Texas, USA

Supplementary Information contains:

Supplementary Figures S1 – S3

Supplementary Tables S1 – S5

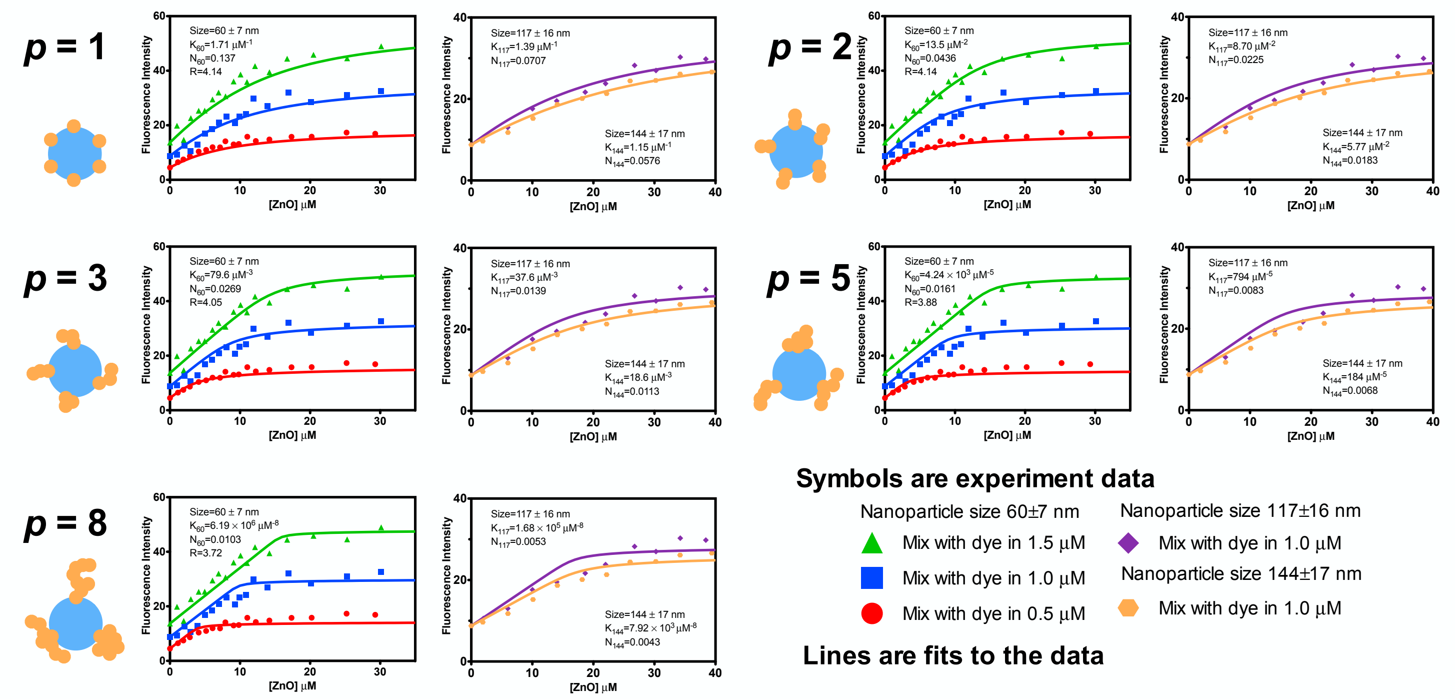

Supplementary Figure S1. Determination of kinetic parameters from spectrofluorometer data. Aqueous suspensions of ZnO nanoparticles (60 ± 7 nm) with initial concentration ranging from 1 to 30 μM were mixed with a fluorescein tracer at concentrations of 0.5, 1.0 and 1.5 μM respectively and analyzed by steady emission scan in a spectrofluorometer. Values of K, N and R yielding best fits to the data were determined at discrete increments of the parameter p. The analysis was repeated to determine parameters corresponding to nanoparticles of different size (117 ± 16 and 144 ± 17 nm).

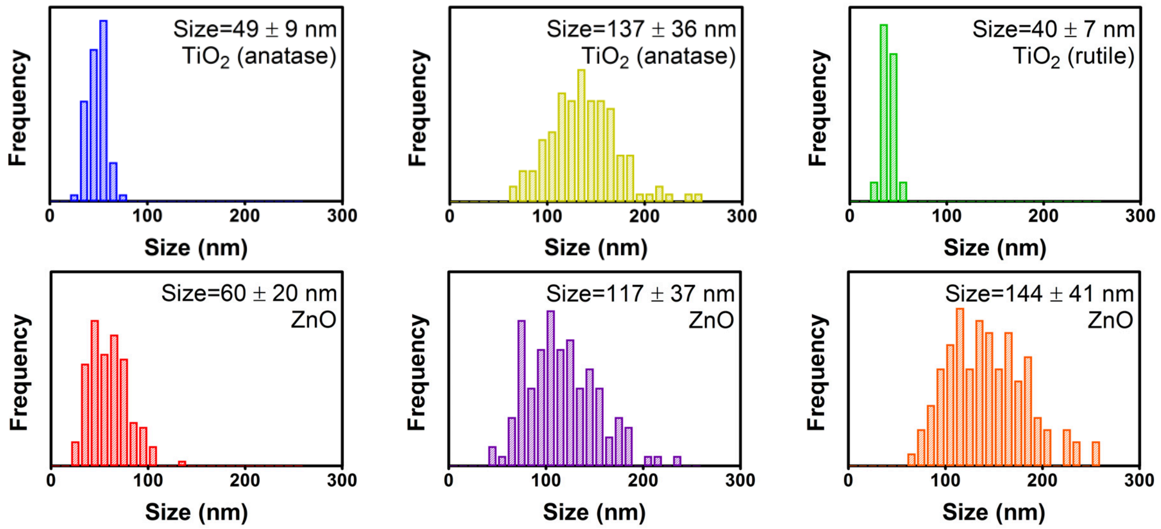

Supplementary Figure S2. **Size distribution data obtained from SEM analysis of nanomaterials studied in the main text.** (a) TiO_2 (anatase), 49 ± 9 nm and (b) 137 ± 36 nm, (c) TiO_2 (rutile), 40 ± 7 nm, ZnO (d) 60 ± 20 nm, (e) 117 ± 37 nm and (f) 144 ± 41 nm.

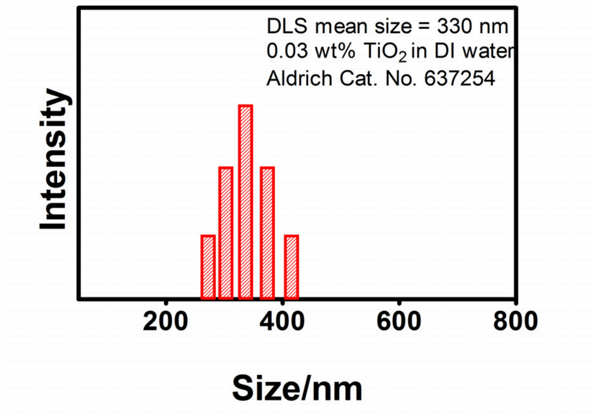

Supplementary Figure S3. **DLS analysis of anatase TiO_2 nanoparticles.** The mean particle size of 49 ± 9 nm obtained via SEM is overestimated by DLS.

Supplementary Table S1a. Summary of kinetic parameters representing ZnO nanoparticles.

	p														
	**1**			**2**			**3**			**5**			**8**		
***d* (nm)**	60	117	144	60	117	144	60	117	144	60	117	144	60	117	144
***N* (mol/mol)**	0.137	0.0707	0.0576	0.0436	0.0225	0.0183	0.0269	0.0139	0.0113	0.0161	0.0083	0.0068	0.0103	0.0053	0.0043
R	4.14			4.14			4.05			3.88			3.72		
***K* (μM^{-p})**	1.71	1.39	1.15	13.5	8.70	5.77	79.6	37.6	18.6	4,240	794	184	6.19×10^{6}	1.68×10^{5}	7.92×10^{3}
**k_{on} (μM^{-p} s^{-1})**	1.71×10^{-2}	1.39×10^{-2}	1.15×10^{-2}	1.08×10^{-2}	6.96×10^{-3}	4.62×10^{-3}	5.57×10^{-3}	2.63×10^{-3}	1.30×10^{-3}	6.36×10^{-4}	3.57×10^{-4}	1.38×10^{-4}	1.86×10^{-5}	5.04×10^{-6}	2.38×10^{-6}
**k_{off} (s^{-1})**	0.01	0.01	0.01	8×10^{-4}	8×10^{-4}	8×10^{-4}	7×10^{-5}	7×10^{-5}	7×10^{-5}	1.5×10^{-7}	4.5×10^{-7}	7.5×10^{-7}	3×10^{-12}	3×10^{-11}	3×10^{-10}
**α ($\times 10^{12}$ m^2/s)**	8.04	4.15	3.38	8.04	4.15	3.38	8.04	4.15	3.38	8.04	4.15	3.38	8.04	4.15	3.38

Supplementary Table S1b. Summary of kinetic parameters employed in simulations involving polydisperse ZnO nanoparticles (parameter values of $p = 5$, $R = 3.88$ used in all calculations).

						**Sample mean size**	
						**60**	**144**
**Size bin (nm)**	**Mean size (nm)**	***N* (mol/mol)**	***K* (μM^{-p})**	**k_{on} (μM^{-p} s^{-1})**	**k_{off} (s^{-1})**	**Particle number %**	**Particle number %**
20-40	30	0.0322	9408	1.64×10^{-3}	1.74×10^{-7}	17.3	0
40-60	50	0.0193	5273	7.59×10^{-4}	1.44×10^{-7}	35.3	0
60-80	70	0.0138	3424	5.82×10^{-4}	1.70×10^{-7}	32.7	3.3
80-100	90	0.0107	2062	5.19×10^{-4}	2.52×10^{-7}	11.3	10.8
100-120	110	0.0088	1060	4.12×10^{-4}	3.89×10^{-7}	2.7	19.2
120-140	130	0.0074	419	2.44×10^{-4}	5.82×10^{-7}	0.7	16.7
140-160	150	0.0064	137	1.14×10^{-4}	8.30×10^{-7}	0	15.8
160-180	170	0.0057	74	8.45×10^{-5}	1.13×10^{-6}	0	15.0
180-200	190	0.0051	51	7.64×10^{-5}	1.49×10^{-6}	0	10.8
200-220	210	0.0046	39	7.43×10^{-5}	1.91×10^{-6}	0	2.5
220-240	230	0.0042	31	7.47×10^{-5}	2.38×10^{-6}	0	4.2
>240	250	0.0039	26	7.65×10^{-5}	2.91×10^{-6}	0	1.7

Supplementary Table S2. Summary of kinetic parameters representing TiO_2 nanoparticles.

	**Anatase TiO_2**	**Rutile TiO_2**
p	1	1
d **(nm)**	49	40
N **(mol/mol)**	0.00025	0.004
R	23	0
K **(μM^{-1})**	1	0.2802
k_{on} **(μM^{-1} s^{-1})**	1	0.5604
k_{off} **(s^{-1})**	1	2
**α (×10^{-12} m^2/s)**	9.9	12.1

Supplementary Table S3. Boundary and initial conditions applied in flow model.

**Laminar flow boundary condition**	
Wall	$\boldsymbol{u} = 0$, $\boldsymbol{n} \cdot \boldsymbol{u} = 0$
Inlet	u = (flow rate) / (cross section area), flow rate = 1.666 e-10 m^3/s
Outlet	$v = 0$, $P = 1$ atm
**Transport boundary condition**	
Wall	$\boldsymbol{n} \bullet \left(-\alpha\nabla[\mathrm{D}] + [\mathrm{D}]\boldsymbol{u}\right) = 0$<br>$\boldsymbol{n} \bullet \left(-\alpha\nabla[\mathrm{N_S}] + [\mathrm{N_S}]\boldsymbol{u}\right) = 0$<br>$\boldsymbol{n} \bullet \left(-\alpha\nabla[\mathrm{ND}] + [\mathrm{ND}]\boldsymbol{u}\right) = 0$
Inlet	[N_S] based on injected suspension concentration and fitted coefficient N<br>[ND] = 0<br>[D] = 0.005 mol/m^3
**Initial values**	
$\boldsymbol{u} = 0$, $P = 1$ atm, [D] = [N_S] = [ND] = 0	

Supplementary Table S4. List of variables and nomenclature.

Variable*	Description
$[N_S]$ (μM)	Available surface binding sites concentration
[D] (μM)	Free dye concentration
[ND] (μM)	Fluorescent complexation concentration
$[M]_i$ (μM)	ZnO concentration calculated by powder mass
p	Bound dye number per surface binding sites
K (μM^{-p})	Equilibrium constant
k_{on} (μM^{-p} s^{-1})	Binding rate constant of dye to surface sites
k_{off} (s^{-1})	Dissociation rate constant of bound dye from surface sites
N (mol/mol)	Coefficient relating ZnO bulk to surface binding sites concentrations
R	Ratio of fluorescence contribution by bound dye to free dye
F_{obs} (a.u.)	Observed fluorescence intensity overall
F'_D (a.u.)	Observed fluorescence intensity by free dye
F'_{ND} (a.u.)	Observed fluorescence intensity by bound dye complexes
F_D (a.u.)	Normalized F'_D
F_{ND} (a.u.)	Normalized F'_{ND}
d (nm)	Nanoparticle diameter
u (m s^{-1})	x-dimension (flow direction) velocity
v (m s^{-1})	y-dimension (lateral direction) velocity
ρ (kg m^{-3})	Density
μ (kg m^{-1} s^{-1})	Viscosity
α (m^2 s^{-1})	Diffusion coefficient

* Subscript *i* refers to variables at initial conditions, while *eq* denotes at equilibrium.

Supplementary Table S5. Summary of nanomaterials and dye employed in our experiments.

Material	Composition	Vendor	Particle size measured by SEM (nm)	Density (g/cm^3)
TiO_2 (anatase)	Powder	Sigma-Aldrich (Cat. No. 637254)	49 ± 9	3.9
TiO_2 (anatase)	Powder	Sigma-Aldrich (Cat. No. 232033)	137 ± 36	3.9
TiO_2 (rutile)	Powder	Sigma-Aldrich (Cat. No. 637262)	40 ± 7	4.17
ZnO	Powder	Sigma-Aldrich (Cat. No. 544906)	60 ± 20	5.6
ZnO	Powder	Sigma-Aldrich (Cat. No. 205532)	117 ± 37	5.6
ZnO	Powder	Sigma-Aldrich (Cat. No. 255750)	144 ± 41	5.6
ZnO	Powder	Alfa-Aesar (Cat. No. 44898)	64 ± 24	5.6
ZnO	Powder	Alfa-Aesar (Cat. No. 44899)	75 ± 33	5.6
ZnO	Powder	Alfa-Aesar (Cat. No. 87812)	155 ± 76	5.6
ZnO	Powder	Alfa-Aesar (Cat. No. 11137)	191 ± 93	5.6
Fluorescein (acid free)	Powder	Fluka Analytical (Cat. No. 46955)	--	--
